# EMG-Triggered Pedaling Training on Muscle Activation, Gait, and Motor Function for Stroke Patients

**DOI:** 10.3390/brainsci12010076

**Published:** 2022-01-04

**Authors:** Kyeongjin Lee

**Affiliations:** Department of Physical Therapy, College of Health Science, Kyungdong University, Wonju 24764, Korea; kjlee@kduniv.ac.kr

**Keywords:** stroke, rehabilitation, electromyography, gait

## Abstract

This study aimed to determine the effects of electromyography (EMG)-triggered pedaling training to improve motor functions in the lower extremities, muscle activation, gait, postural balance, and activities of daily living in stroke patients. Subjects were randomly allocated to two groups: the EMG-triggered pedaling training group (EMG-PTG, *n* = 21) and the traditional pedaling training group (TPTG, *n* = 20). Both groups trained five times per week for four weeks, with 50 min per session. Lower extremity motor function was assessed using the Fugl–Meyer Assessment (FMA). Muscle activation of the four muscles of the lower extremities was assessed using eight-channel electromyography, while gait ability was assessed using GaitRite. Postural balance was assessed using the Berg balance scale (BBS), the timed up and go (TUG), and functional reach tests (FRT). Daily activities were assessed using the Modified Barthel Index (MBI). For lower extremity motor function, gait ability, balance ability, and activities of daily living, the EMG-PTG showed significant improvement compared to TPTG (*p* < 0.05). These results suggest that EMG-triggered pedaling training effectively improves lower extremity motor function, muscle activation, gait, postural balance, and activities of daily living in stroke patients.

## 1. Introduction

The number of stroke patients has increased as a result of an increase in the survival rate of acute stroke as well as an aging population [1]. Quality of life declines as a consequence of impaired physical function. [2,3]. Hemiparesis is one major cause of gait impairments, and therefore, restoring gait function is one of the most important goals for stroke rehabilitation [4].

Stroke gait is characterized by asymmetric movement and compensatory gait patterns due to weakness on one side of the body [5]. Asymmetric gait patterns can be identified through an analysis of spatial-temporal gait variables, including step length, swing, and stance phase durations [6]. The duration of the swing phase decreases as the stance phase extends. Hemiplegic gait presents with excessive hip joint and pelvic movements in order to extend the decreased step length [7]. Prolonged and repeated compensatory gaits result in serious disability, which leads to limitations in mental and social participation and an eventually cause a decline in a stroke patient’s quality of life [8]. Clinicians and researchers have been testing many treatment strategies to reduce gait asymmetry, and their results have been recorded in previous studies [6,9,10].

Constraint-induced movement therapy (CIMT) involves forcefully restraining the unaffected side to induce the use of the affected side [11]. It originally began with the concept of forcefully restraining the unaffected side to activate the affected limbs. However, more recent studies have suggested the concept of using the affected limb actively to create purposeful movement by repetitively practicing concrete movements [12]. CIMT has been reported to be effective in restoring upper limb function by inducing changes in brain function and structure [13]. CIMT has been mostly used in the rehabilitation of motor function in the upper limbs, but few have used it for the lower extremities [14]. The use of CIMT has been more favorable in upper limb rehabilitation because most daily activities that are performed with the upper limbs can be executed with only one side, making it much easier to forcefully restrain the other side. Meanwhile, the lower extremities are mostly employed for locomotion, and gait—its representative function—is impossible with only one leg. Therefore, applying CIMT to the lower extremities is unfavorable. However, recent studies have adopted CIMT to the lower extremities by placing a shoe insert on the unaffected side [9], using a knee orthosis [15], or attaching weight to the unaffected ankle [16]. Their efforts have enhanced balance ability and gait speed and have facilitated weight loading on the affected side [9,16,17].

Stationary bicycles are widely used in stroke rehabilitation [18]. In addition, they can be suggested to patients with insufficient balance, as they only require moving the legs alternatively while sitting on a stable chair [19]. This method has the advantage of being simple to use and can enhance motor control, endurance, balance, and cardiopulmonary function [20,21]. Pedaling exercises on stationary bicycles can be divided into flexion and extension phases. The flexion phase activates the knee flexors and ankle dorsiflexors, whereas the extension phase activates the knee extensors and ankle plantar flexors. This activation pattern is similar to the muscle pattern that is activated during gait. Therefore, these bicycles can be used for gait training preparation or for those who have insufficient functional ability to train with reciprocal movement patterns [22].

Stroke patients tend to depend on their unaffected side, reducing the stance phase and step length of the affected side [23]. Moreover, they mostly use their unaffected side during pedaling, reducing the opportunity to train the affected side. Voluntary movement has an integral influence on the motor cortex and the supplementary motor area of the brain [24]. Therefore, there is a need for strategies to train the muscles on the affected side. Electromyography (EMG)-triggered stimulation, which is one of the methods used to induce voluntary movement of the affected side, has been reported to enhance voluntary contraction and the gait ability of the affected side by activating the motor cortex of the brain by concentrating on cognitive function and eccentric motor nerve recruitment [25].

In this study, we attached EMG electrodes to the affected lower limb muscles and developed an EMG-triggered pedaling device that moved when the voluntary contraction of the affected lower limb was present to evaluate the effect of EMG-triggered pedaling training on gait ability, postural balance, lower limb strength, functional recovery, and activities of daily living (ADL) in stroke patients.

## 2. Materials and Methods

### 2.1. Subjects

The subjects who were involved in this study were recruited from inpatient stroke patients at D Hospital in Seoul, Korea, with the following inclusion criteria: (1) chronic hemiplegic stroke patients who had been diagnosed with a stroke for more than six months, (2) patients with sufficient cognitive function (mini-mental state examination over 24 points), and (3) a Brunnstrom motor recovery stage that was higher than the third stage. The exclusion criteria included having any neurological deficits other than stroke, orthopedic problems, including fracture or peripheral nerve injury, visual problems or deficits, auditory problems, history of more than double stroke, and a participation of less than 80% during the experiment. Prior to the experiment, the purpose and procedure of the study were explained to all of subjects, and all of them provided written consent. All of the experimental procedures in this study were approved by the Institutional Review Board.

To calculate the sample size, G-power 3.19 software (Heinrich Heine University Düsseldor, Düsseldorf, Germany) was used with a significance level of 0.05 and a power of 0.8. The effect size was calculated as 0.93, which was based on the gait speed variables from a pilot study. The sample size per group was 20, and after considering dropouts, 22 subjects were recruited per group.

### 2.2. Experimental Procedure

Of the 60 stroke patients who recruited, 44 were selected based on the inclusion and exclusion criteria. Before the experiment, 16 patients who did not meet the criteria were excluded from the screening test by a doctor. Five of them were excluded because they scored lower than 24 points on the Mini-Mental State Exam (MMSE), and the other 11 could not walk independently for more than 10 m. After the screening, the participants were randomly allocated into an EMG-triggered pedaling training group (EMG-PTG) or a traditional pedaling training group (TPTG). To minimize allocation bias, a random allocation software (version1.0) (M. Saghaei, Isfahan, Iran) was used, and sex, side of paresis, cause, duration of stroke, and cognitive ability were randomly distributed to ensure homogeneity. Both groups had underwent five training sessions per week for four weeks, and each session was 50 min long. Prior to the experiment, general characteristics, gait ability, postural balance, lower limb strength, functional recovery, and ADL were evaluated.

**Figure 1:**
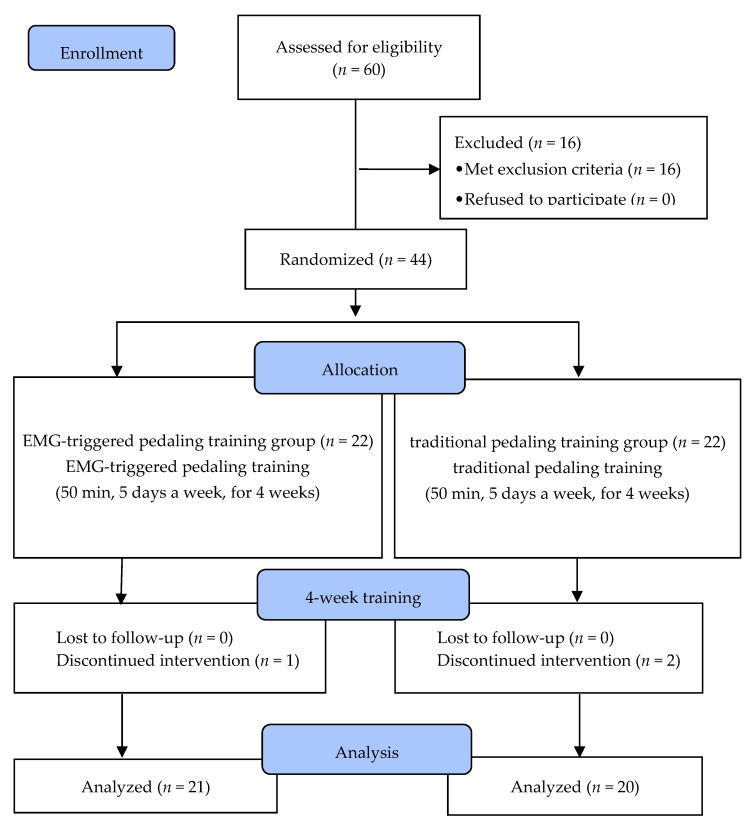
Flow diagram of the study. EMG, Electromyography.

During the intervention period, subjects who could not persist with the program because of alternations in medical conditions, and those who participated in less than 80% of the whole program were excluded from the final study. In the EMG-PTG, one patient was discharged during the experiment, and two patients from the TPTG (traditional pedaling training group) were dropped because they participated in less than 80% of the program. Ultimately, 21 subjects in the EMG-PTG and 20 in the TPTG participated in the study. All subjects underwent pre- and post-tests without exception, and the data from these tests were statistically analyzed (Figure 1).

### 2.3. Experimental Method

#### 2.3.1. EMG-Triggered Device

**Figure 2:**
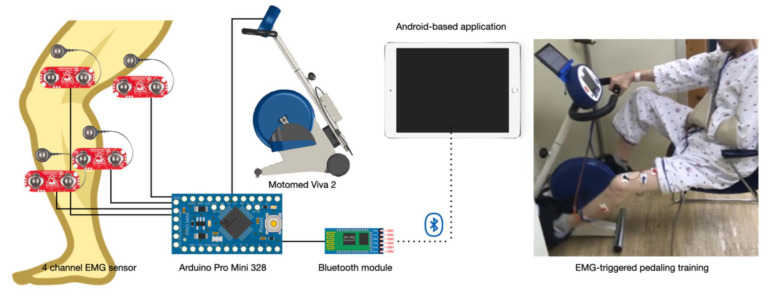
EMG-triggered pedaling training device.

A training device was developed for the EMG-triggered pedaling training. An application was developed to control a stationary bicycle by integrating signals from a 4-channel EMG sensor. The device was composed of three parts: an EMG sensor receiver, a sensor-connected stationary bicycle, and a table with the controlling application. The EMG sensor receiver collected muscle activation signals from four EMG electrodes (MyoWare Muscle Sensor, SparkFun Electronics, Niwot, CO, USA) that were located on the rectus femoris, biceps femoris, tibialis anterior, and gastrocnemius muscles. The signals were processed into packets using Arduino Pro Mini 328 (Arduino Pro Mini 328, SparkFun Electronics, Niwot, CO, USA), and the processed signals were transferred to the table through Bluetooth (Figure 2). The receiver was installed inside the control panel of a stationary bicycle.

The Android-based application was able to visualize and control the signals from the receiver. To set the EMG baseline threshold, the subjects sat on the chair of the bicycle and pedaled at a comfortable speed, while the EMG electrodes (Ag/AgCl surface electrode H2223H, 3M, Maplewood, MN, USA) were placed on the lower limb muscles of the affected side. The activity of the four muscles was measured for 30 s and was analyzed with the application, and the average of the activity was calculated and used as the baseline threshold to increase the speed of the bicycle. The sum of the activity signals from the four muscles was used to calculate the average value, and the same process was used during the training period. After the threshold was set, the subject pedaled until they reached their threshold, at which point, the speed of the bicycle increased; when they did not reach the threshold, the speed decreased. The baseline value to increase the speed initially was set at 150% of the average activation, and a therapist could manually change the value by 10% depending on the condition of the subjects. In the control application, bar graphs were displayed, and the patients could watch the graphs during training. A stationary bicycle (Motomed Viva 2, RECK-Technik GmbH & Co, Betzenweiler, Germany) has been developed by RECK-Technik to rehabilitate patients with neurological disabilities. This MOTOmed system can be used independently, both at medical facilities and home. The user can be trained in motions that are similar to those for cycling. The system was adapted clinically in a phased rehabilitation program for hemiplegic stroke patients. It provides various modes to support functional recovery in a safe environment with smooth movement.

#### 2.3.2. EMG-Triggered Pedaling Training

A 10 min warm-up training exercise was conducted at the patient’s desired speed prior to the main training session to ensure that the training was safe. After the warm-up, the 30 s baseline measurement was conducted to set the EMG threshold. The subjects started training at their preferred speed, and the speed was changed repetitively during the session depending on the goal set by the therapist. The main exercise lasted for three sessions of 10 min each, with a one-minute break between each session. After the main exercise, a five-minute cool-down exercise was also conducted at the patient’s preferred speed. The therapist monitored the subjects during training. Subjects who complained of dizziness or difficulty during the training rested immediately before training again. Subjects were told that they could stop the training any time they wanted. The TPTG was trained on the same bicycle but without the EMG-triggered device in passive mode and with the same amount of training time. The EMG-triggered training proceeded after any ankle foot orthoses that the patients used during their normal gait were removed.

### 2.4. Outcome Measurements

#### 2.4.1. Muscle Activation

Surface electromyography (Ultium EMG^®^, Noraxon, Scottsdale, AZ, USA) with dual EMG wet gel electrodes (single electrode T246H, SEEDTECH, Gyeonggi, Korea) was used to measure muscle activation. All of the electrodes were placed after shaving and abrading the skin with alcohol. The position of the electrodes was as follows: rectus femoris, 50% on the line from the anterior iliac spine superior to the superior part of the patella; biceps femoris, 50% on the line between the ischial tuberosity and the lateral epicondyle of the tibia; tibialis anterior, 1/3 on the line between the tip of the fibula and the tip of the medial malleolus; gastrocnemius, 1/3 of the line between the head of the fibula and the heel. The sample rate was 2000 Hz with a band filter of 20–400 Hz and notch filters of 60 Hz. Raw data were processed into root mean square (RMS) values with a window of 60 ms after rectification and smoothing. In surface electromyography, the current that was generated during muscle contraction was amplified through a total of eight wireless transmitters and was delivered to the receiver to collect muscle activity. The collected data were stored and were analyzed using electromyography software (myoRESEARCH MR 3.5.4, Noraxon Inc., Scottsdale, AZ, USA).

The symmetry ratio was used to determine the degree of left-right symmetry during the pedaling exercise in the subjects [6]. It is also a value that represents the symmetry between the paralyzed and non-paralyzed sides. A symmetry ratio value of 1 indicates that both sides are completely symmetric and can be calculated using the following formula (Equation (1)):

(1) $$\mathrm{Symmetry}\;\mathrm{ratio}=\frac{\mathrm{unaffected}\;\mathrm{side}\;\mathrm{limb}}{\mathrm{affected}\;\mathrm{side}\;\mathrm{limb}}$$

Muscle activity was measured during a pedaling assessment using an EMG sensor connected to a bicycle with the resistance intensity set to zero. The pedaling direction was clockwise, and the highest position of the right pedal was set to zero. The pedaling was performed at the patient’s preferred speed in a comfortable position by placing the distance between the bicycle and seat such that the knee angle could be between 135° and 150°, while the angle between the bicycle and pedal was 90°. The subject had enough time to practice getting used to pedaling. The pedaling assessment was performed for one minute, and all of subjects wore short and loose pants to prevent interruptions.

#### 2.4.2. Gait Ability

The spatiotemporal parameters of gait were assessed using a gait assessment device (GAITRite, CIR System Inc., Franklin, NJ, USA) to collect qualitative analysis data of the gait patterns of the subjects [26,27]. A GAITRite is a device with an intra-rater reliability of r = 0.90, and an inter-rater correlation coefficient (ICC = 0.99) of r = 0.96 [28,29]. General gait variables such as velocity and cadence, temporal variables such as stride and step time, single support and double support percentage of the gait cycle, and spatial variables such as step and stride lengths were recorded after a computerized analysis. The device is 5 m in length, 61 cm in width, and 0.6 cm in height and contains 16,128 sensors that are 1 cm in size and that are arranged 1.27 cm apart vertically along the gait plate to collect spatiotemporal variables. For the assessment, the subject stood in front of the gait plate and then walked with a comfortable velocity through the plate upon verbal command from a therapist. In this study, each subject walked comfortably to measure all of the spatiotemporal gait variables and used their usual supportive devices. All of the assessments were randomly measured three times, and the average value for each test was used.

Spatiotemporal variables are integral to confirming gait symmetry among stroke patients. In this study, we used the temporal and spatial ratio used by that Patterson et al. [8] to calculate the gait symmetry ratio. The equation for the symmetry ratio is shown in (Equations (2) and (3)). This study calculated the symmetry ratios of step length, step time, and single-limb stance time using the temporal and spatial symmetry ratios. The absolute values were used to calculate the symmetry. A value that is close to zero means increased gait symmetry, while a value that is further from zero indicates decreased symmetry.

(2) $$\mathrm{Spatial}\;\mathrm{symmetry}=1\;-\frac{\mathrm{unaffected}\;\mathrm{side}\;\mathrm{step}\;\mathrm{length}}{\mathrm{affected}\;\mathrm{side}\;\mathrm{step}\;\mathrm{length}}$$

(3) $$\mathrm{Temporal}\;\mathrm{symmetry}=1\;-\frac{\mathrm{unaffected}\;\mathrm{side}\;\mathrm{step}\;\mathrm{length}\;\mathrm{time}}{\mathrm{affected}\;\mathrm{side}\;\mathrm{step}\;\mathrm{length}\;\mathrm{time}}$$

#### 2.4.3. Lower Extremity Motor Function

Lower extremity motor function for this study was assessed using the Fugl–Meyer Assessment Lower Extremity (FMA-LE) scale, which evaluates functional recovery in stroke patients. Each item of the test is rated on a three-point scale: 0 points for inability to proceed, 1 point for partial completion, and 2 points for the full completion of a task. The maximum score for lower limb function was 34 points, and the categories for the test consisted of hip, knee, ankle joints, and coordination. Aside from these, balance, sensation, and pain assessment are also available, but only the scores for lower limb function were used in this study. The test has excellent interrater (r = 0.94) and intra-rater (r = 0.99) reliabilities [30].

### 2.5. Data Analysis

The Statistical Package for the Social Sciences (SPSS version 19, IBM, Armonk, NY, USA) software was used for all of the statistical analyses of the data. Normality was checked with the Shapiro–Wilk test, and the mean and standard deviation were calculated. Data were normally distributed, and the sociodemographic characteristics of the subjects were analyzed with real numbers, percentages, means, and standard deviations. Independent t-tests and chi-square tests were used to test the homogeneity between the two groups. The changes in the dependent variables were analyzed with a paired t-test, and the effect between groups was calculated using an independent t-test. The effect size was calculated to evaluate the strength of the training effect, and the minimal detectable change (MDC)—which reflects the true change in addition to errors—was also calculated. The statistical significance level(α) was set as 0.05 for all of the available data.

## 3. Results

### 3.1. General Characteristics of the Subjects

The general characteristics of the participants are listed in Table 1. There were no notable differences between the general characteristics of the two groups, including age, height, weight, body mass index, duration of stroke, mini-mental state examination score, modified Barthel index score, sex, paretic side, and stroke type.

### 3.2. Changes of in Muscle Activation

The changes in the muscle activation for both groups after training are shown in Table 2. The changes in muscle activation fir the affected side and the symmetry ratio in the rectus femoris, biceps femoris, and gastrocnemius muscles showed significant improvement in EMG-PTG after the training (*p* < 0.05), whereas TPTG showed no significant changes. A comparison of the groups based on the training method showed significant improvements in the EMG-PTG in relation to the TPTG (*p* < 0.05).

### 3.3. Changes of Gait Ability

The changes in the gait parameters for both groups after training are shown in Table 3. Temporal gait parameters, including velocity, cadence, stride time, and double-limb support time, improved significantly in both groups after the training (*p* < 0.05), while the affected side step time and single-limb support time only improved in the EMG-PTG (*p*< 0.05). A comparison of the groups based on the training method showed significant improvements in velocity, affected side step time, single-limb support time, and double-limb support time in the EMG-PTG (*p* < 0.05) in relation to the TPTG.

Spatial gait parameters, including stride length and affected side step length significantly improved in the EMG-PTG after the training period (*p* < 0.05), whereas the TPTG showed no significant changes. A comparison of the groups according to the training method showed significant improvements in the EMG-PTG in relation to the TPTG (*p* < 0.05). In addition, the step length of the unaffected side significantly improved in both groups (*p* < 0.05). However, when comparing the groups according to the training method, the EMG-PTG was significantly improved compared to the TPTG (*p* < 0.05).

Among the symmetry of gait variables, the gait symmetry of step length and step time significantly improved in the EMG-PTG after the training period (*p* < 0.05), but the TPTG showed no statistically significant changes. In addition, a comparison of the groups according to the training method showed significant improvements in gait symmetry on step time in the EMG-PTG (*p* < 0.05) as opposed to in the TPTG.

### 3.4. Changes of Lower Extremity Motor Function

The changes in the motor function in the lower extremities after training in each group are shown in Table 4. The results of FMA-LE showed statistically significant improvements in both groups (*p* < 0.05). However, the EMG-PTG showed a significantly higher improvement compared to the TPTG.

## 4. Discussion

This study aimed to adapt a pedaling exercise to train the basic gait pattern by inducing a spontaneous contraction of the affected side and to evaluate the effect of this exercise on gait ability, postural balance, lower limb muscle activation, functional recovery, and ADL. Pedaling training has been used extensively to improve the gait ability of stroke patients, as it utilizes muscle patterns that are similar to those of the lower limb and presents reciprocal crossing movement and is therefore considered to be appropriate for training to adapt to gait changes [18,22,31].

Pedaling training has many functional advantages, but the mechanisms underlying these effects are still unclear. In addition to this, the question of whether the improved spatiotemporal gait variables were definitely due to improvements in the recovery of the affected side was raised. Rehabilitation of the lower limb involves the use of both legs, which can create a higher dependence on the unaffected side. In the short term, compensation using the unaffected side during pedal training may increase the patient’s functional status. In addition, complete passive pedal training makes it difficult to actively participate with the affected side [32].

EMG-triggered pedaling training, which was developed in this study, is more meaningful because it supplements these defects and induces active participation of the affected side. To induce active spontaneous contraction of the affected side, we analyzed the muscle activity in the affected side during pedaling and developed a device that only increases the speed when the signal exceeds a preset threshold value. Biofeedback from the muscle activity in the target muscle group were provided to induce participation and motivation from the patient. Bolton et al. [33] reported that initiating voluntary movement activates cognition in the motor cortex and complementary motor areas.

In this study, gait variables were analyzed after dividing them into temporal and spatial variables, after which their symmetry was evaluated. Common characteristics of gait in stroke patients include reduction in gait speed, cadence, stride length, step length, the single-limb support phase, the double-limb support phase, and step time [6,34].

The EMG-PTG showed a significant improvement in the symmetry of the temporal and spatial variables. While there was no significant difference in step time on the unaffected side, the step time on the affected side showed a significant decrease of 11.6%, and the temporal and spatial symmetry of the TPTG did not change. After the intervention, a large effect size based on Cohen’s effect size analysis was present, with an effect size of 0.93 for step length and 0.80 for the step time symmetries. These results show that the rate of minimum detectable change (MDC%) for the step length symmetry exceeded 91.3% (MDC 0.10), and for step time symmetry, it exceeded 88.8% (MDC 0.03). This means that the swing phase speed of the affected side increased during the single-limb support phase of the unaffected side in the EMG-PTG. The step length significantly improved on both the affected and unaffected sides. The effect size of step length for the affected side showed a large effect size of 0.91, and on the unaffected side, there was a median effect size of 0.65 and an increase of 10.2%. These results met the MDC for the spatial gait variables with significant results. This may indicate an improvement in the weight-bearing ability of the affected side. Both groups showed improvements in gait ability, but the EMG-PTG showed a significant improvement in gait, with an increase in velocity of 22.4%, an effect size of 1.26, and a significant MDC% of 35.71% (MDC 2.56).

Previous studies using pedaling training have reported improved gait ability [26]. However, the most important consideration is how much of the change was made on the affected side. In a study by Kautz et al. [32], the effect of pedaling training on the muscle activity of the lower extremities was observed through EMG, but there was no significant changes that were on the affected side. A recent study on cycling in stroke patients reported methods that combined functional electrical stimulation with pedaling [27,35]. Functional electrical stimulation showed partial improvements in some gait variables but did not present any significant differences overall; therefore, functional electrical stimulation did not lead to significant improvements on the affected side [10]. While functional electrical stimulation therapy is a passive method, the EMG-triggered pedaling training used in this study can be described as an active training method because it allows movement training by maximizing the residual function, as shown in the results of this study. Yang et al. [20] reported significant results in functional recovery and gait ability in the experimental group in a study evaluating the effect of biofeedback cycling training. They used a pedaling training method that provided visual biofeedback on a monitor regarding the loads applied on both legs and that spontaneously contracted in accordance with a preset speed. Jung et al. [36] conducted gait training by inducing increased weight bearing on the affected side by providing a cane that gave auditory feedback on the activation of the paralyzed lower extremity. The experimental group showed significant increases in muscle activity and gait speed on the affected side, and their stance phase increased. This is thought to have improved gait by limiting movement on the unaffected side and providing re-learning, symmetry, and balance on the lower limb of the affected side through proprioceptive and motor stimulation from simultaneous biofeedback and voluntary movement. This result is important because it emphasizes the active movement of the affected side to harmonize with the unaffected side [14].

In stroke patients, postural balance and gait are impaired due to an imbalance in the muscle strength and activity between the affected and unaffected sides, which eventually affects their daily activities [37]. During pedaling training, the knee extensors and flexors, dorsiflexors, and plantar flexors are mainly functional [31]. In this study, we attempted to reduce asymmetry through a training method that induces the spontaneous contraction on the affected side. Before training, asymmetry between the sides was present, with the activity of the unaffected side being approximately twice that of the affected side. After the training, the EMG-PTG improved muscle activity on the affected side, except for the tibialis anterior, and decreased the asymmetry in the muscle activity between the unaffected and affected sides. However, the TPTG did not exhibit any significant changes. This difference is thought to be caused by the induced voluntary contraction of the affected side in the EMG-PTG, and the improvement in the muscle activity of the affected side may have had a positive effect on the improvements seen in gait and balance. Stroke patients present a marked decrease in knee flexion and dorsiflexion. In the results of this study, the improvement in the knee flexors and dorsiflexors was lower than that of the knee extensors and plantar flexors, and there were no statistically significant improvements in the dorsiflexors. The effect size was calculated to be 0.68–1.13, which is a high effect size that meets the MDC for the change in the lower extremity muscle activity of the affected side, with a significant result.

The lower extremity motor function was examined with the Fugl–Meyer test and was used to evaluate the changes that could be observed after the training period and in the recovery of movement; here, the EMG-PTG showed significant improvement. Karthiga [38] conducted pedaling training by providing visual biofeedback to 20 stroke patients. As a result, it was shown that the motor function of the lower extremities in the experimental group was significantly improved in the Fugl–Meyer lower extremity scale and in the step test scores. The FMT-LE evaluates voluntary movements and active contractions of the hip, knee, and ankle joints. This test is consistent with the purpose of the training, which was to induce spontaneous contractions. Previous studies on EMG biofeedback training and constraint-induced motion therapy have been reported to improve functional recovery by activating the muscles of the paralyzed limb [16,24,39]. Concentrating on the use of the affected side through cognitive function and somatosensory biofeedback from the movement of the affected side through EMG-triggered pedaling training based on the concepts of EMG biofeedback and lower limb constraint-induced movement therapy may have activated the cerebral motor cortex to induce alterations in muscle activation.

Based on the results of this study, we believe that EMG-triggered pedaling training improved gait, balance, lower extremity muscle activity, and functional recovery, leading to improvement in the ADL of stroke patients. The results of this study are limited to stroke patients who have residual function in their affected lower limbs. Another limitation is that the persistence of the effect could not be confirmed because no follow-up study was conducted.

## Figures and Tables

**Table 1:** General characteristics of the subjects.

	EMG-PTG (*n* = 21)	TPTG (*n* = 20)	χ^2^/*t*	*p*
Age (year)	62.71 ± 6.91	64.80 ± 9.59	0.802	0.428
Height (cm)	162.62 ± 7.85	161.70 ± 9.33	0.342	0.734
Weight (kg)	59.10 ± 8.17	59.93 ± 8.53	0.319	0.752
Body mass index (point)	22.30 ± 2.27	22.85 ± 1.93	0.828	0.413
Duration of stroke (month)	13.71 ± 5.97	16.70 ± 5.83	1.619	0.113
MMSE	25.86 ± 1.31	25.50 ± 1.05	0.958	0.344
MBI	53.10 ± 10.00	56.01 ± 10.05	0.929	0.359
Gender (male/female)	10/11	10/10	0.023	0.879
Paretic side (right/left)	10/11	14/6	2.114	0.146
Stroke type (infarction/hemorrhage)	14/7	12/8	0.196	0.658

Values are expressed as mean ± standard deviation. The independent *t*-test and chi-squared tests are used to compare the dependent variables between the two groups. EMG-PTG, electromyography-triggered pedaling training group; TPTG, traditional pedaling training group; MMSE, mini-mental state examination; MBI, modified Barthel index.

**Table 2:** Changes of muscle activation.

		EMG-PTG (*n* = 21)	TPTG (*n* = 20)	Significance of Change Scores *t(p)*	Effect Size	MDC (MDC%)
Muscle activation of affected side						
Rectus femoris muscle (μV)	Pre	7.58 ± 4.26	7.08 ± 4.02			
	Post	10.19 ± 4.60	7.88 ± 4.68			
	Change Score	2.61 ± 1.35 *	0.80 ± 2.03	3.364(0.002)	1.05	0.82(31.35)
Biceps femoris muscle (μV)	Pre	12.00 ± 5.09	12.13 ± 4.91			
	Post	13.73 ± 5.22	12.84 ± 5.75			
	Change Score	1.73 ± 0.74 *	0.70 ± 2.00	2.192(0.034)	0.68	0.45(25.84)
Tibialis anterior muscle (μV)	Pre	11.16 ± 9.18	13.83 ± 10.12			
	Post	11.90 ± 9.63	14.71 ± 9.47			
	Change Score	0.73 ± 1.82	0.87 ± 2.13	0.224(0.824)		
Gastrocnemius muscle (μV)	Pre	14.05 ± 12.65	12.79 ± 11.76			
	Post	15.84 ± 12.73	13.45 ± 11.80			
	Change Score	1.79 ± 1.27 *	0.67 ± 1.84	2.278(0.028)	0.71	0.77(42.85)
Muscle activation of unaffected side						
Rectus femoris muscle (μV)	Pre	12.48 ± 5.18	12.76 ± 6.27			
	Post	12.79 ± 4.93	12.90 ± 5.38			
	Change Score	0.19 ± 1.64	0.14 ± 1.90	0.056(0.932)		
Biceps femoris muscle (μV)	Pre	23.57 ± 15.82	20.73 ± 15.75			
	Post	24.08 ± 15.71	21.36 ± 15.82			
	Change Score	0.51 ± 1.98	0.62 ± 2.17	0.175(0.862)		
Tibialis anterior muscle (μV)	Pre	18.19 ± 9.91	18.95 ± 9.05			
	Post	18.60 ± 10.12	19.54 ± 9.38			
	Change Score	0.40 ± 1.75	0.59 ± 1.99	0.320(0.751)		
Gastrocnemius muscle (μV)	Pre	15.25 ± 5.22	14.29 ± 5.12			
	Post	15.59 ± 5.60	14.84 ± 5.02			
	Change Score	0.34 ± 1.62	0.55 ± 2.17	0.363(0.718)		
Symmetry ratio						
Rectus femoris muscle	Pre	1.97 ± 0.89	2.12 ± 1.01			
	Post	1.40 ± 0.64	2.04 ± 1.10			
	Change Score	−0.57 ± 0.52 *	−0.08 ± 0.80	2.343(0.024)	0.73	0.32(55.33)
Biceps femoris muscle	Pre	2.00 ± 1.06	1.67 ± 0.94			
	Post	1.76 ± 0.91	1.69 ± 1.00			
	Change Score	−0.24 ± 0.26 *	0.03 ± 0.21	3.630(0.001)	1.13	0.16(65.38)
Tibialis anterior muscle	Pre	2.13 ± 1.18	1.77 ± 0.87			
	Post	2.12 ± 1.39	1.67 ± 0.75			
	Change Score	−0.01 ± 0.57	−0.10 ± 0.35	0.605(0.549)		
Gastrocnemius muscle	Pre	1.71 ± 1.10	1.66 ± 1.10			
	Post	1.42 ± 0.88	1.78 ± 1.39			
	Change Score	−0.29 ± 0.30 *	0.12 ± 0.52	3.073(0.004)	0.96	0.18(62.46)

Values are expressed as mean ± standard deviation. * means significant difference within groups. EMG-PTG, electromyography-triggered pedaling training group; TPTG, traditional pedaling training group; MDC, minimal detectable change.

**Table 3:** Changes of gait ability.

		EMG-PTG (*n* = 21)	TPTG (*n* = 20)	Significance of Change Scores *t(p)*	Effect Size	MDC(MDC%)
Temporal gait parameter						
Velocity (cm/s)	Pre	32.40 ± 16.41	33.71 ± 15.23			
	Post	39.66 ± 18.83	36.35 ± 16.75			
	Change Score	7.26 ± 4.29 *	2.64 ± 2.91 *	4.018(0.000)	1.26	2.56(35.71)
Cadence (step/min)	Pre	56.75 ± 14.36	57.73 ± 13.92			
	Post	63.94 ± 18.80	61.22 ± 17.52			
	Change Score	7.18 ± 6.58 *	3.49 ± 5.55 *	1.936(0.060)		
Stride time (s)	Pre	2.34 ± 1.05	2.30 ± 1.07			
	Post	2.15 ± 1.11	2.22 ± 1.11			
	Change Score	−0.19 ± 0.22*	−0.08 ± 0.14 *	2.005(0.052)		
Affected side step time (s)	Pre	1.32 ± 0.54	1.32 ± 0.54			
	Post	1.17 ± 0.55	1.29 ± 0.55			
	Change Score	−0.15 ± 0.13 *	−0.03 ± 0.11	3.128(0.003)	0.98	0.08(52.11)
Unaffected side step time (s)	Pre	1.02 ± 0.52	0.98 ± 0.54			
	Post	0.98 ± 0.56	0.94 ± 0.59			
	Change Score	−0.04 ± 0.12	−0.04 ± 0.10	0.184(0.855)		
Affected side Single-limb support time (s)	Pre	0.41 ± 0.14	0.42 ± 0.14			
	Post	0.44 ± 0.14	0.41 ± 0.15			
	Change Score	0.03 ± 0.05 *	−0.01 ± 0.07	2.568(0.014)	0.80	0.03(88.80)
Unaffected side Single-limb support time (s)	Pre	0.54 ± 0.13	0.58 ± 0.14			
	Post	0.54 ± 0.14	0.58 ± 0.16			
	Change Score	0.00 ± 0.04	0.01 ± 0.07	0.112(0.912)		
Double-limb support (%)	Pre	55.82 ± 14.82	53.04 ± 13.73			
	Post	49.11 ± 16.27	51.63 ± 12.84			
	Change Score	−6.71 ± 3.32 *	−1.42 ± 2.98 *	5.359(0.000)	1.67	2.01(29.93)
Spatial gait parameter						
Stride length (cm)	Pre	66.12 ± 22.79	68.43 ± 21.17			
	Post	72.10 ± 20.61	69.47 ± 19.12			
	Change Score	5.98 ± 5.53 *	1.04 ± 3.66	3.354(0.002)	1.05	3.34(55.88)
Affected side Step length (cm)	Pre	35.52 ± 13.51	36.45 ± 12.79			
	Post	38.37 ± 12.24	36.26 ± 10.90			
	Change Score	2.86 ± 2.76 *	−0.19 ± 3.88	2.913(0.006)	0.91	1.67(58.38)
Unaffected side Step length (cm)	Pre	30.60 ± 9.57	31.97 ± 8.70			
	Post	33.73 ± 8.90	33.21 ± 8.70			
	Change Score	3.12 ± 3.50 *	1.24 ± 2.08*	2.084(0.044)	0.65	2.12(67.84)
Symmetry of gait						
Gait symmetry on step length (score)	Pre	0.20 ± 0.09	0.18 ± 0.10			
	Post	0.16 ± 0.12	0.18 ± 0.17			
	Change Score	−0.04 ± 0.08 *	0.00 ± 0.17	0.963(0.341)		
Gait symmetry on step time (score)	Pre	0.34 ± 0.18	0.41 ± 0.27			
	Post	0.24 ± 0.12	0.50 ± 0.45			
	Change Score	−0.10 ± 0.16 *	0.09 ± 0.25	2.971(0.005)	0.93	0.10(91.13)

Values are expressed as mean ± standard deviation. * means significant difference within group. EMG-PTG, electromyography-triggered pedaling training group; TPTG, traditional pedaling training group; MDC, minimal detectable change.

**Table 4:** Changes in lower extremity motor function.

		EMG-PTG (*n* = 21)	TPTG (*n* = 20)	Significance of Change Scores *t(p)*	Effect Size	MDC (MDC%)
FMA-LE (score)	Pre	15.90 ± 4.71	15.08 ± 4.13			
	Post	19.62 ± 5.12	15.84 ± 3.17			
	Change Score	3.72 ± 1.53 *	0.76 ± 3.17 *	6.419(0.000)	2.01	0.93(24.92)

Values are expressed as mean ± standard deviation. * means significant difference within group. EMG-PTG, electromyography-triggered pedaling training group; TPTG, traditional pedaling training group; MDC, minimal detectable change; FMA-LE, Fugl-Meyer assessment-lower extremity.

## Data Availability

Informed consent was obtained from all subjects involved in the study.
